# Complete polarization characterization of single plasmonic nanoparticle enabled by a novel Dark-field Mueller matrix spectroscopy system

**DOI:** 10.1038/srep26466

**Published:** 2016-05-23

**Authors:** Shubham Chandel, Jalpa Soni, Subir kumar Ray, Anwesh Das, Anirudha Ghosh, Satyabrata Raj, Nirmalya Ghosh

**Affiliations:** 1 Department of Physical Sciences, Indian Institute of Science Education and Research Kolkata, Mohanpur- 741246, India

## Abstract

Information on the polarization properties of scattered light from plasmonic systems are of paramount importance due to fundamental interest and potential applications. However, such studies are severely compromised due to the experimental difficulties in recording full polarization response of plasmonic nanostructures. Here, we report on a novel Mueller matrix spectroscopic system capable of acquiring complete polarization information from single isolated plasmonic nanoparticle/nanostructure. The outstanding issues pertaining to reliable measurements of full 4 × 4 spectroscopic scattering Mueller matrices from single nanoparticle/nanostructures are overcome by integrating an efficient Mueller matrix measurement scheme and a robust eigenvalue calibration method with a dark-field microscopic spectroscopy arrangement. Feasibility of *quantitative Mueller matrix polarimetry* and its potential utility is illustrated on a simple plasmonic system, that of gold nanorods. The demonstrated ability to record full polarization information over a broad wavelength range and to quantify the *intrinsic plasmon polarimetry* characteristics via Mueller matrix *inverse* analysis should lead to a novel route towards quantitative understanding, analysis/interpretation of a number of intricate plasmonic effects and may also prove useful towards development of polarization-controlled novel sensing schemes.

Optical properties of noble metal nanoparticles/nanostructures, governed by the so-called surface plasmon resonance (SPR) effects have evoked intensive investigations in recent times owing to their fundamental nature and potential applications^1^,^2^. The SPR can be of two types- propagating at metal-dielectric interfaces, or localized in the case of metal nanoparticles/nanostructures. The localized plasmon resonances, owing to their distinctive spectral (wavelength dependent) characteristics and inherent sensitivity towards local dielectric environment, are being pursued for numerous practical applications. The applications include, biomedical and chemical sensing, bio-molecular manipulation, contrast enhancement in optical imaging, surface enhanced spectroscopy, development of novel nano-optical devices, optical information processing, data storage, developing polarization measurement devices using plasmonic particles, plasmonic metasurfaces and so forth^1^,^2^,^3^,^4^,^5^,^6^,^7^,^8^,^9^,^10^,^11^,^12^,^13^,^14^,^15^. Besides the potential applications, a number of interesting and intricate fundamental effects associated with the interaction of light with specially designed plasmonic nanostructures have also been observed recently. Spin orbit interaction (SOI) and Spin Hall (SH) effect of light^16^,^17^, Plasmonic Aharonov-Bohm effect^18^, optical analogue of quantum weak measurements in plasmonic systems^19^, quantum spin hall effect^20^, Goos–Hänchen (GH) and Imbert–Fedorov (IF) shifts in plasmonic structures^21^, spin controlled plasmonics^22^, coupled plasmons and plasmonic Fano resonances^23^,^24^,^25^, are some of the recently discovered plasmonic effects having fundamental consequences in diverse areas ranging from quantum, atomic to condensed matter systems. Knowledge on the polarization properties of the scattered light is crucial for fundamental understanding of the aforementioned effects because polarization plays an important role in the light-matter interactions leading to most (if not all) of these effects. Moreover, the polarization information should also prove useful for optimizing experimental parameters for many practical applications. For instance, this can be exploited to develop polarization-controlled novel schemes for contrast enhancement in biomedical imaging and for optimizing/enhancing sensitivity of plasmonic sensors^3^. Although, some inroads in ‘*plasmon polarimetry*’ has already been made, these are usually limited to measurements involving excitation with selected state of polarization and subsequent detection of the corresponding co- and/or the crossed polarized components of the scattered light^26^,^27^. Such approaches, limited by their framework of obtaining partial information on the polarization transfer, have only proven moderately successful in selected applications for empirically extracting semi-quantitative information on the underlying complex nature of the polarized light-matter interactions^23^,^26^,^27^. But overall, the full potential of *quantitative polarimetry* in the context of plasmonics is yet to be realized. Our theoretical investigations have indicated that recording of full spectral Mueller matrices should prove to be extremely valuable in this regard^28^. Mueller matrix is a 4 × 4 matrix representing the transfer function of any optical system in its interaction with polarized light and all the medium polarization properties are characteristically encoded in its various elements. Recently, such Mueller matrix measurements have been performed in the reflection geometry from plasmonic crystal sample (large scale periodic array of metallic nanostructures), and analysis/interpretations of the matrix elements were done via the Fresnel reflection coefficients and conventional ellipsometry parameters^29^. Recording of Mueller matrix from plasmonic nanostructures/nanoparticles exclusively using the scattered light and its inverse analysis via the scattering polarimetry parameters, on the other hand, should provide new insights and enable quantitative analysis/interpretation of a number of intricate fundamental effects in plasmonic systems. Once recorded, the scattering Mueller matrix can be analyzed to extract/quantify the intrinsic polarization properties of the medium, namely, *diattenuation* (differential attenuation of orthogonal polarization states either by scattering or by absorption) and *retardance* (phase difference between orthogonal polarizations)^25^. These Mueller matrix-derived parameters may potentially be used to probe and quantify the relative strengths and phases of the interfering plasmon modes in complex coupled plasmonic systems such as those exhibiting plasmonic Fano resonance, analyze/interpret SOI and Spin Hall effect, GH and IF shifts mediated by scattering from plasmonic systems and so forth^21^,^28^,^30^.

Despite the wealth of interesting effects that can be probed using spectral scattering Mueller matrices of plasmonic nanostructures/nanoparticles, its experimental realization remains to be an outstanding challenge. The challenges include: (1) the scattering signal from plasmonic nanostructures is rather weak and is often swamped by the large background unscattered light, (2) recording of full Mueller matrix over a broad wavelength range simultaneously in combination with the corresponding spatial maps (spectral Mueller matrix images) by itself is a formidable task, (3) this is confounded further by the fact polarimetric measurements on plasmonic nanostructures often necessitates use of high numerical aperture (NA) microscopic setting, (4) challenges in analysis and quantification of the measured polarization signals or images and complexities in understanding and interpreting the plasmon polarimetry results. In order to address these outstanding challenges, in this paper, we present a novel experimental system that integrates a Mueller matrix measurement scheme with dark-field microscope to record full 4 × 4 spectroscopic scattering Mueller matrices from a single isolated nanoparticle/nanostructure. The dark field microscopic arrangement facilitates detection of weak scattering signal from the plasmonic nanostructures and has been previously explored for scattering spectroscopic studies on nanoparticles/nanostructures^31^,^32^,^33^. Here, we integrate an efficient Mueller matrix measurement scheme and a robust polarization calibration method with the dark-field microscopic spectroscopy arrangement to facilitate recording of complete polarization response of a single nanoparticle. Note that there are inherent complexities of polarization measurements over broad spectral range due to the wavelength dependent polarization response of the optical components. Moreover, for polarization measurement in high NA geometry, one needs to account for the polarization transformation due to the focusing and collection geometry. These problems are systematically dealt with a robust eigenvalue calibration method. This approach enabled determination and incorporation of the exact experimental polarization responses of the polarization state generator/analyzer units (including the effect of the high NA imaging geometry) over the wavelength range of interest. The developed approach thus facilitates recording of the spectral polarization response (spectroscopic Mueller matrix) exclusively of the plasmonic system with desirable accuracy. The experimental polarimetry system is complemented with Mueller matrix *inverse* analysis models to tackle the issues on analysis and quantification of the measured polarization signals from plasmonic systems. Initial exploration of this ‘*comprehensive plasmon polarimetry platform*’ on gold nanorods demonstrates the promise of the Mueller matrix-derived plasmon polarimetry parameters as novel experimental metrics for studying a number of interesting plasmonic effects. An illustrative example is presented on how such information can be utilized to probe, manipulate, and controllably tune the interference of the neighboring resonant modes (orthogonal electric dipolar modes in plasmonic nanorods) and the resulting spectral line shape of the plasmonic system via polarization control.

## Results and Discussion

**Figure 1:**
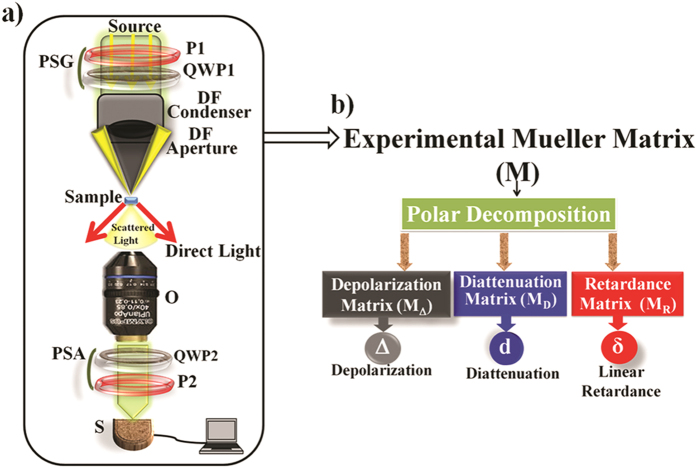
A schematic of the comprehensive plasmon polarimetry platform. (**a**) Dark-field Mueller matrix spectroscopic microscopy system: White light from the mercury lamp after passing through the PSG unit, is focused to an annular shape at the sample site using a dark-field (DF) condenser. PSG: Polarization state generator, PSA: Polarization state analyzer. (P1, P2): fixed linear polarizers, (QWP1, QWP2): achromatic quarter waveplates. The sample-scattered light is collected by an objective and passed through the PSA unit for spectrally resolved signal detection, performed by a spectrometer (S). The 4 × 4 spectral scattering Mueller matrices are constructed using sixteen measurements performed with sixteen optimized combinations of PSG and PSA units. (**b**) The Mueller matrix *inverse* analysis models enable decomposition of the experimental Mueller matrix into basis matrices of depolarizing (depolarization matrix) and non-depolarizing (diattenuation and retardance matrices) effects for subsequent extraction/quantification of the constituent polarimetry parameters.

A *comprehensive plasmon polarimetry platform*, comprising of (a) dark-field Mueller matrix spectroscopic microscopy experimental system and (b) Mueller matrix *inverse* analysis models(shown in Fig. 1), was explored to record and analyze the spectroscopic (wavelength λ = 400–700 nm) scattering Mueller matrices *M*(λ) from single isolated plasmonic nanoparticle/nanostructure and other samples. The results of these studies are presented here. Specifically, we *(i)* briefly describe the Mueller matrix construction scheme and the *inverse* analysis approach, *(ii)* present the results of system calibration and *(iii)* describe the results of spectral Mueller matrix measurements and *inverse* analysis on single isolated plasmonic gold (Au) nanorod samples. The potential utility of such quantitative Mueller matrix polarimetry on plasmonic system is also illustrated and discussed in this context.

### Scheme for Mueller matrix construction

The 4 × 4 spectral Mueller matrices were constructed by combining sixteen spectrally resolved intensity measurements (spectra) for four different combinations of the optimized elliptical polarization state generator (using the PSG unit) and analyzer (using the PSA unit) basis states (see Fig. 1a and Experimental Materials and Methods). The four elliptical polarization states are generated by sequentially changing the fast axis of QWP1 to four angles (*ϑ* = 35°, 70°, 105° and 140°) with respect to the axis of P1. These four sets of Stokes vectors (4 × 1 vector) are grouped as column vectors to form the 4 × 4 generator matrix*W*. Similarly, the four elliptical analyzer basis states are obtained by changing the fast axis of QWP2 to the corresponding four angles (35°, 70°, 105° and 140°). The analyzer states are analogously written as a 4 × 4 analyzer matrix *A*. The sixteen sequential intensity measurements (at any wavelength) are grouped in a 4 × 4 matrix *M*_*i*_, which is related to *A*, *W* matrices and the sample Mueller matrix *M* as,




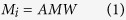




The Mueller matrix *M* can be determined using known forms of the *A* and *W* matrices as




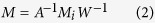




The optimal generator and the analyzer basis states were obtained via optimization of the ratio of the smallest to the largest singular values of the individual square matrices *W*and *A*. The orientation angles of the quarter waveplates (*ϑ* = 35°, 70°, 105° and 140°) were decided based on this^34^. In principle, *M* can be determined from experimental *M*_*i*_, by using theoretical forms of *W*and *A* matrices (obtained by using the standard Mueller matrices of the polarizer and the quarter waveplates). However, this is confounded by – (1) the complex nature of the polarization transformation due to the high NA imaging geometry leading to significant changes in the *W*(λ) and *A*(λ) matrices; (2) the *W*(λ) and *A*(λ) matrices may vary significantly with wavelength due to the non-ideal behavior of the polarizing optics over the wavelength range. We tackled these issues using a robust eigenvalue calibration method, which takes care of the spectral polarization responses of the polarization state generator, analyzer units, the focusing and the detection systems (imaging geometry) by determining the exact experimental forms of the *W*(λ) and *A*(λ) matrices^35^. Using this approach, the *W*(λ) and *A*(λ) matrices are determined by performing measurements on a set of ideal calibrating samples (pure diattenuators (polarizers) and retarders (waveplates)). The specifics of the various steps of this method can be found elsewhere (see Supplementary information)^34^,^35^. Note that on conceptual ground, Mueller matrix is ideally defined for plane waves. However, for practical purposes, such measurements can be extended towards low/high NA imaging geometry, where excitation is done using finite beams (even paraxial beams represent spread in excitation angle) or focused light and the resulting sample-scattered light is collected over a finite detection angle. In such situations, if the polarization transformation due to the imaging geometry (focusing and collection) is appropriately accounted for, the resulting scattering Mueller matrix should still encode exclusive information on the sample scattering polarimetry effects integrated over the excitation and the scattering angles. Since, in our approach, the polarization transformation due to the high NA imaging geometry is taken care by using the eigenvalue calibration method, this approach facilitated recording of the spectral polarization response (Mueller matrix) exclusively of the plasmonic and other scattering samples. The effect of the excitation/collection geometry on the Mueller matrix-derived polarimetry parameters of our plasmonic (Au nanorod) samples are discussed subsequently.

### Mueller matrix inverse analysis

In general, plasmonic samples may exhibit all the polarimetry effects, namely, diattenuation, retardance and depolarization. These may contribute in a complex interrelated way to the Mueller matrix elements, masking potentially interesting information and hindering their interpretation. This issue was addressed by performing *inverse* analysis on the experimental Mueller matrices using Polar decomposition method, which is a reliable and widely used approach for solving the *inverse* problem in polarimetry^36^. Using this approach, the experimental Mueller matrix is decomposed into three basis matrices corresponding to the three elementary polarimetry effects




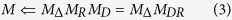




Here, *M*_*Δ*_, *M*_*R*_ and *M*_*D*_ describe the depolarizing effects, the retardance effect (both linear and circular), and the diattenuation effect (linear and circular) of the medium, respectively (shown in Fig. 1b). The latter two are combined as the ‘non-depolarizing’ diattenuating retarder Mueller matrix *M*_*DR*_ here. Note that the scattering matrix (defined in a given scattering plane) for regular shaped scatterer is generally non-depolarizing and represents a preferentially oriented diattenuating retarder Mueller matrix for a chosen scattering plane (specified by an azimuthal angle of scattering)^30^. However, when excitation and collection of scattered light is performed over a range of angles (as in high NA imaging geometry), incoherent addition of the individual (corresponding to each azimuthal and polar angle of scattering) non-depolarizing Mueller matrices leads to the depolarization effect (arises due to orientation averaging of diattenuating retarder Mueller matrices, as discussed subsequently). In our approach, we have therefore filtered out this depolarized component (where the intrinsic sample polarization information is scrambled) via *M*_Δ_ in Eq. 3, and extracted the polarized component of the scattered light (via *M*_*DR*_). The intrinsic sample polarization information is therefore contained in the resulting (non-depolarizing) diattenuating retarder matrix *M*_*DR*_, which is subjected to further analysis (results presented subsequently).

Once decomposed, the constituent polarimetry parameters depolarization (Δ), linear diattenuation (*d*) and linear retardance (*δ*) are quantified as^36^




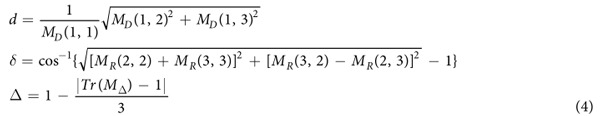




Note that we have not considered circular diattenuation and circular birefringence effects (which can also be extracted from the decomposed matrices) due to the *achiral* nature of our samples. The accuracy of spectral Mueller matrix measurements in high NA microscopic setting and the ability to quantify the constituent polarimetry effects from experimental Mueller matrices were first tested on calibrating sample exhibiting diattenuation and retardance effects. Following successful calibration, this was explored on single isolated plasmonic gold (Au) nanorod samples, as described subsequently.

### System calibrations results

**Figure 2:**
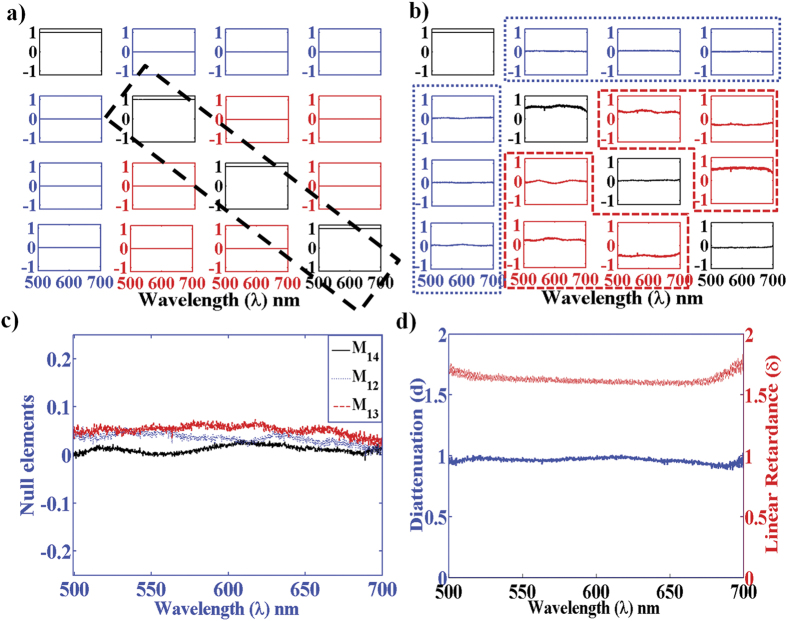
Results of eigenvalue calibration of the dark-field Mueller matrix spectroscopic microscopy system. (**a**) The experimental spectral (λ = 500–700 nm) Mueller matrices for blank (with no sample) nearly resemble the identity matrices. (**b**) The spectral Mueller matrices of a calibrating achromatic quarter waveplate exhibit characteristic behaviour of linear retarder associated with symmetries in the off-diagonal elements of the lower 3 × 3 block (highlighted in redcolour). The Mueller matrices in (**a,b**) are shown in normalized unit (normalized by the element *M*_11_(λ)). (**c**) The expected null elements of the quarter waveplate (the elements of the first row shown here). (**d**) The Mueller matrix-derived (using Eq. 4) linear retardance *δ*(λ) (right axis, reddotted line) and linear diattenuation *d*(λ) (left axis, bluesolid line) of the achromatic quarter waveplate and a linear polarizer, respectively.

The results of the eigenvalue calibration on reference samples (linear polarizer as pure diattenuator and achromatic quarter waveplate as pure retarder) are summarized in Fig. 2. Note that there are practical limitations in performing the eigenvalue calibration on the transparent (non-scattering) reference samples because one can only detect the sample-scattered light in the exact dark-field configuration. To allow the detection of signal from the reference samples which are non-scattering elements (a polarizer and a quarter wave plate) in our case, the collection objective was kept marginally away from the exact focus thus allowing a very weak leakage signal in the form of an annular ring from the dark field aperture to be detected by the spectrally resolved detection unit. This approach adequately incorporated the wavelength dependent response of the polarizing optical components and the polarization transformation of the high NA imaging geometry, by determining the actual experimental *W*(λ) and *A*(λ) matrices, as demonstrated in Fig. 2. The blank (with no sample) Mueller matrices constructed using the experimental *W*(λ) and *A*(λ) matrices in Eq. 2, nearly resemble identity matrices (Fig. 2a, off-diagonal elements are nearly zero, ≤0.05) over the wavelength range (λ = 500–700 nm, shown here). The Mueller matrices of the achromatic quarter waveplate (Fig. 2b) exhibit the expected behavior of a pure linear retarder over the entire λ-range (characterized by significant magnitudes of the elements of the lower 3 × 3 block along with the associated symmetries in the elements). Further, the expected null elements of the retarder (the elements of the first row and the first column) are also nearly zero (Fig. 2c, elemental error ≤0.05). The values for linear retardance (*δ*) and linear diattenuation (*d*) of the quarter waveplate and the linear polarizer (respectively) were determined from their experimental Mueller matrices (using Equation. 4) and are shown in Fig. 2d. The derived magnitudes of 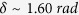 and *d* ~ 0.98 (over λ = 500–700 nm) are in good agreement with the expected ideal values (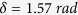 for quarter waveplate and *d* = 1 for linear polarizer). Based on these and calibration studies on various other reference samples, the measurement strategy appears valid for performing accurate sample Mueller matrix measurements despite numerous complexities associated with the high NA microscopic imaging geometry that too over a broad wavelength range. The ability to record full 4 × 4 spectroscopic Mueller matrices in the dark-field configuration and to quantify the *intrinsic* sample polarimetry characteristics bodes well for quantitative polarimetric investigations on plasmonic nanoparticles/nanostructures, which is our primary goal.

### Mueller matrix studies on plasmonic Au nanorods

**Figure 3:**
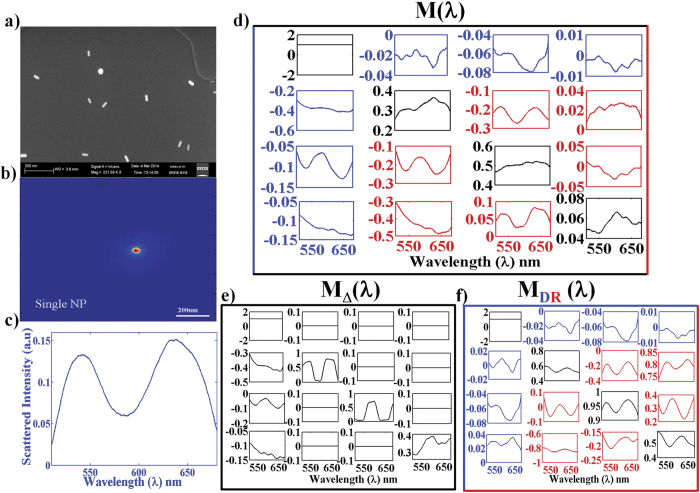
Results of spectroscopic Mueller matrix measurements on single isolated Au nanorod. (**a**) SEM image of Au nanorods. (**b**) Dark-field image of a single Au nanorod. (**c**) Typical scattering spectra (with un-polarized excitation, corresponding to *M*_11_(λ) element) recorded from the Au nanorod exhibits two distinct peaks corresponding to the two (transverse and longitudinal) electric dipolar plasmon resonances. (**d**) The spectral scattering Mueller matrices *M*(λ) of the Au nanorod exhibit characteristic features of the constituent elementary polarimetry properties - depolarization reflected in the diagonal elements (black); diattenuation in the first row and column (blue); linear retardance in the off-diagonal elements of the lower 3 × 3 block (red). (**e,f**) The wavelength dependence of the decomposed(using Eq. 3) basis matrices encoding the depolarizing (in depolarization matrix −*M*_Δ_(λ)) and the non-depolarizing (in diattenuating retarder matrix-*M*_*DR*_(λ)) effects. All the matrices are shown in normalized unit (normalized by *M*_11_(λ)).

**Figure 4:**
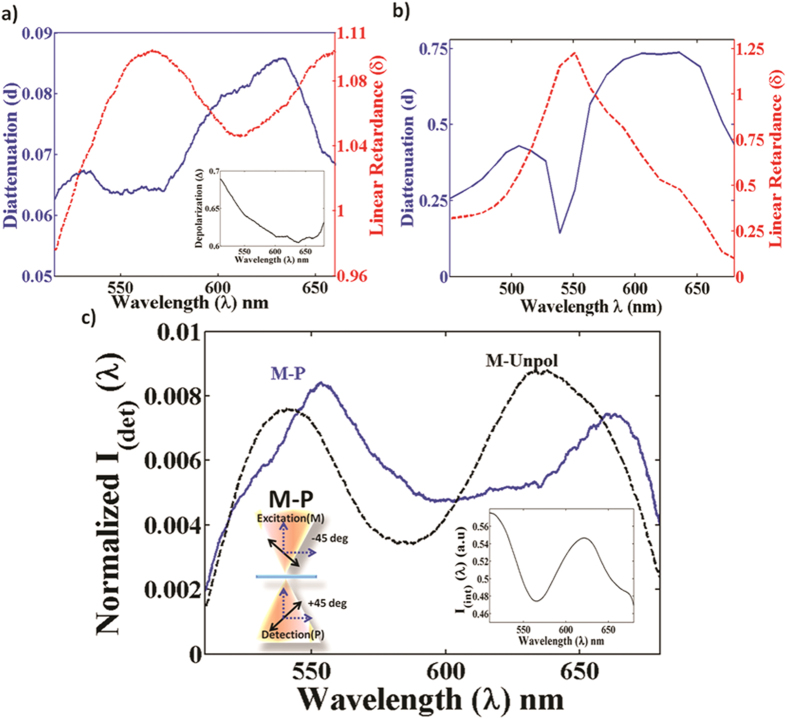
The results of the *inverse* analysis on the spectral scattering Mueller matrices *M*(λ) of the Au nanorod (corresponding to Fig. 3). (**a**) The Mueller matrix-derived wavelength variation of the linear retardance *δ*(λ) (right axis, red dotted line) and linear diattenuation *d*(λ) (left axis, blue solid line) parameters. The inset shows wavelength variation of the decomposition-derived depolarization coefficient Δ(λ). (**b**) The corresponding theoretically computed *d*(λ) and *δ*(λ) parameters for a preferentially oriented similar Au nanorod, under plane wave excitation. (**c**) The spectral line shapes of the scattered intensity *I*_*det*_(λ) for excitation with −45° linear polarization (M) and subsequent detection using +45° (P) analyzer basis (blue solid line) and without any analyzer (polarization-blind detection, black dotted line). The strength of the interference signal (*I*_*int*_(λ)) is displayed in the inset, wherein a cartoon of the polarization state generator and the analyzer is also displayed.

Figure 3 displays the results of spectroscopic Mueller matrix measurements on single isolated Au nanorod. The Au nanorods were chemically synthesized following the procedure described in Experimental Materials and Methods^37^. The average dimensions of the Au nanorods were as follows: diameter = 14 ± 3 nm, length = 40 ± 3 nm, aspect ratio (ratio of diameter to length) ε ~ 0.35 (determined from the SEM image, Fig. 3a). The solution containing the Au nanorods was diluted adequately so that there was a single isolated nanorod in the field of view of the dark-field microscopic arrangement (shown in Fig. 3b). Typical scattering spectra recorded from the Au nanorod (Fig. 3c) exhibits two distinct peaks corresponding to the two electric dipolar plasmon resonances, one at shorter λ (transverse resonance along the short axis at λ ~ 525 nm) and the other at longer λ (longitudinal resonance along the long axis at λ ~ 650 nm)^38^. The corresponding scattering Mueller matrices *M*(λ) exhibit several interesting spectral trends (Fig. 3d). The complicated nature of *M*(λ) with essentially all sixteen non-zero matrix elements underscore the fact that even a single isolated plasmonic Au nanorod exhibit all the elementary polarimetry characteristics (diattenuation, retardance and depolarization), thus highlighting the need for Mueller matrix *inverse* analysis. The considerably low magnitudes of the diagonal elements (*M*_*22*_*, M*_*33*_ and *M*_*44*_) imply overall strong depolarizing nature of the scattered light. Non-zero intensities of the elements in the first row and the first column (*M*_*12*_*/M*_*21*_*, M*_*13*_*/M*_*31*_), on the other hand, is a manifestation of the linear diattenuation effect. Strong signature of the linear retardance effect is also evident from significant intensities of the off-diagonal elements in the lower 3 × 3 block of *M*(λ) (*M*_*34*_/*M*_*43*_ and *M*_*24*_*/M*_*42*_ representing retardancefor horizontal/vertical and +45°/−45° linear polarizations respectively). The spectral trends of the effects are gleaned further and become more evident in the decomposed (using Equation. 3) basis matrices, the depolarization effect in the matrix −*M*_Δ_(λ) (Fig. 3e) and the diattenuation and retardance effects in the non-depolarizing matrix-*M*_*DR*_(λ) (Fig. 3f). The observed linear diattenuation in the plasmonic nanorods originates from the differential excitation of the two orthogonal dipolar plasmon resonances (transverse and the longitudinal) by orthogonal linear polarizations. Linear retardance, on the other hand, is a manifestation of the inherent phase retardation between the two competing dipolar plasmon modes^28^. As previously discussed, that under plane wave excitation, the scattering Mueller matrix from preferentially oriented plasmonic nanorod should be non-depolarizing (diattenuating retarder) in nature. However, unlike ideal plane wave excitation, here the nanorod is excited by focused light beam and the scattered light is also detected in high NA imaging geometry (using an objective lens). This leads to orientation averaging (orientation of the axis of the rod with respect to the illuminating polarization) and averaging over several forward scattering angles (decided by the NA of the objective) and scattering planes (azimuthal angles of scattering). The resulting incoherent addition of diattenuating retarder Mueller matrices (representing intensities corresponding to the individual constituent polarization preserving scattered fields) eventually manifest as depolarization effect. It is evident from the magnitudes of the diagonal elements of the decomposed depolarization matrix *M*_Δ_(λ) (Fig. 3e) that despite the high NA imaging geometry, ~30–40% of the detected light is still polarized (on an average the polarized fraction is ~[1 − Δ], with maximum magnitude of Δ(λ) ~ 0.65, see subsequent Fig. 4a). The resulting (non-depolarizing) diattenuating retarder matrix *M*_*DR*_ (which contains intrinsic sample polarization information) can therefore be used to reliably extract the scattering-induced diattenuation and the retardance effects, and may also be relevant for further analysis on the polarized component of the scattered light.

Interestingly, despite such high NA imaging geometry, the intrinsic spectral characteristics of the linear diattenuation and the retardance effects of single plasmonic nanorod are preserved. This is illustrated in Fig. 4a, wherein the decomposition-derived (using Equation. 4) linear diattenuation *d*(λ) and linear retardance *δ*(λ) parameters are displayed. Whereas the magnitude of *d*(λ) peaks at the wavelengths corresponding to the two orthogonal dipolar plasmon resonances (λ ~ 525 nm for the transverse and ~650 nm for the longitudinal), the magnitude of *δ*(λ) attains its maximum value at the spectral overlap region of the two resonances (λ ~ 575 nm). For comparison, the theoretically computed (using T-matrix approach^39^) scattering Mueller matrix-derived *d*(λ) and *δ*(λ) parameters for a preferentially oriented Au nanorod (having similar dimension as the experimental one) under plane wave excitation, are displayed in Fig. 4b. The spectral behavior of the experimental *d*(λ) and *δ*(λ) parameters are in good agreement to the corresponding theoretical predictions. The corresponding wavelength dependence of the derived depolarization coefficient Δ(λ) is shown in the inset of Fig. 4a. The observed intriguing spectral diattenuation and retardance effects can be interpreted via the relative amplitudes and the phases of the two orthogonal dipolar plasmon polarizabilities of the Au nanorod. In the dipole approximation (valid for scatterer dimension a ≪ *λ*), the two orthogonally polarized amplitude scattering matrix elements (representing the scattered fields) of the nanorod can be modeled as *s*_*l*_ ∝ *α*_*l*_ cos *θ* and *s*_*t*_ ∝ *α*_*t*_, where *α*_*l*_ and *α*_*t*_ are the longitudinal and the transverse dipolar plasmonpolarizabilities, respectively, and *θ* is the scattering angle^38^. The diattenuation *d* and the linear retardance *δ* parameters are linked to the two competing resonant polarizabilities as^28^




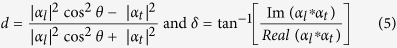




As apparent from Eq. 5, the magnitude of *δ* is directly related to the phase difference between the two orthogonal dipolar plasmon polarizabilities (longitudinal *α*_*l*_ and transverse *α*_*t*_). The diattenuation, on the other hand, is primarily determined by the relative strengths of the two orthogonal dipolar plasmon polarizabilities (|*α*_*l*_| and |*α*_*t*_|). Information on the relative strengths and phase differences between the two resonant plasmon polarizabilities are therefore characteristically encoded in the *d*(λ) and *δ*(λ) parameters, respectively. Note that in the dipolar approximation, the magnitude of *δ* does not depend on the polar scattering angle *θ* or angle of excitation (exclusively related to the phase difference between the two intrinsic plasmon modes). The magnitude of the parameter *d* would however, weakly depend on the scattering angle *θ* also (thus on the choice of the excitation/scattering direction) because of the cos^2^ *θ* factor associated with dipolar scattering. In conformity with this, while the computed *δ* parameters for the Au nano-rod shows no angular dependence, the magnitude of *d* varies weakly with increasing forward scattering angle *θ* (see the computed angular dependence of the *d* and *δ* parameters for Au nano-rod, Supplementary Figure S1). The magnitude of the derived *δ*(λ) parameter is accordingly reasonably close to the theoretical prediction for preferentially oriented Au nano-rod under plane wave excitation (Fig. 4a,b). The magnitude of the *d*(λ) parameter, on the other hand, is lower than the corresponding theoretical predictions (Fig. 4a,b), as averaging over forward scattering angles affects this parameter. Importantly, the characteristic plasmonic spectral behaviour of the *d*(λ) and *δ*(λ) parameters are retained because these are directly linked to the relative strengths of the orthogonal dipolar plasmon polarizabilities and phase difference between them, respectively.

The ability to capture and quantify unique information on the relative strengths and the phases of the contributing plasmon resonance modes via the wavelength dependence of the *d*(λ) and *δ*(λ) parameters may open-up interesting new avenues for the analysis/interpretation of the interference of the neighboring modes in coupled plasmonic structures. An illustrative example of this for the Au nanorod is demonstrated in Fig. 4c (corresponding to the Mueller matrices of Fig. 3f). The results are displayed for excitation with −45° linear polarization (denoted by M) and subsequent detection of the scattered light intensity (*I*_*det*_(λ)) with +45° analyzer basis state (denoted by P) and that without any analyzer (polarization-blind detection). For the sake of comparison, the spectral line shapes of *I*_*det*_(λ) (normalized by the total intensity 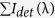) rather than the absolute values of the detected intensities are shown (as expected for the polarization preserving component of the scattered light, the detected cross-polarized M-P component was much weaker in intensity than the polarization-blind detected M-Unpol component). Polarization blind detection leads to no interference effect of the two plasmon resonance modes, leading to mere addition of scattered intensities of the two modes (the detected signal is typically *I*_*det*_(λ) ~ |*s*_*l*_(λ)|^2^ + |*s*_*t*_(λ)|^2^). Projection of the scattered light into +45° linear polarization basis detection, on the other hand, leads to contribution of the interference signal [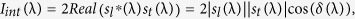 as encoded in the Mueller matrix elements *M*_33_(λ)/*M*_44_(λ)] in addition to the scattered intensity contributions of the two individual plasmon modes [|*s*_*l*_(λ)|^2^ + |*s*_*t*_(λ)|^2^]. The appearance of the interference signal leads to distinct changes in the spectral line shape, manifesting as an asymmetry in the resulting line shape (Fig. 4c). The corresponding strength of the interference signal and its wavelength dependence *I*_*int*_(λ) is separately shown (in relative units) in the inset of Fig. 4c. These results initially demonstrate the potential utility of the spectral scattering Mueller matrices *M*(λ) and the derived *d*(λ) and *δ*(λ) polarization parameters for probing, manipulating and controllably tuning the spectral interference effect in a simplest possible plasmonic system. Its potential applications on more complex coupled plasmonic structures (such as those exhibiting plasmonicFano resonances^20^,^21^,^22^) can now be envisaged.

## Conclusions

To summarize, a novel experimental system is developed for the recording of full 4 × 4 spectroscopic scattering Mueller matrices from single isolated plasmonic nanoparticle/nanostructure. The system overcomes the outstanding issues pertaining to reliable measurements of weak scattering polarization signals from nanoparticle/nanostructures over broad spectral range that too in high NA imaging geometry, by integrating an efficient Mueller matrix measurement scheme and a robust eigenvalue calibration method with a dark-field microscopic spectroscopy arrangement. Feasibility of *quantitative Mueller matrix polarimetry* using the developed system is illustrated on a simple plasmonic system, that of gold nanorods. To the best of our knowledge, this is the first ever report on quantitative Mueller matrix polarimetry on plasmonic systems. The results revealed intriguing spectral diattenuation *d*(λ) and retardance *δ*(λ) effects from single isolated plasmonic Au nanorod, as quantified via Mueller matrix inverse analysis. It is demonstrated further that these Mueller matrix-derived plasmon polarimetry parameters, *d*(λ) and *δ*(λ) encode potentially valuable information on the relative strengths (amplitudes) and phases of competing neighboring resonant modes in plasmonic structures. These polarimetry parameters therefore hold considerable promise as novel experimental metrics for the analysis/interpretation of a number of interesting plasmonic effects; for instance, these can be used to probe, manipulate and tune the interference of the neighboring modes in complex coupled plasmonic structures, to study SOI^30^, Spin Hall effect and other polarization-dependent shifts in plasmonic structures^21^, to optimize/develop polarization-controlled novel plasmonic sensing schemes, and so forth. We are currently expanding our investigations in these directions. In general, the unprecedented ability to record full polarization information over a broad wavelength range and to quantify the intrinsic polarimetry characteristics from even a single isolated nanoparticle/nanostructure may prove useful for spectro-polarimetric characterization of a wide class of complex nano materials.

## Experimental Materials and Methods

### Mueller matrix experimental system

The experimental system (shown in Fig. 1a) is capable of acquiring full 4 × 4 spectroscopic scattering Mueller matrices from single isolated plasmonic nanoparticle/nanostructure over a broad wavelength range (λ = 400–700 nm). The system essentially comprises of three units- conventional inverted microscope (IX71, Olympus) operating in the dark-field imaging mode, polarization state generator (PSG) and polarization state analyzer (PSA) units, and spectrally resolved signal detection (spectroscopy) unit. Collimated white light from a mercury lamp (U-LH100L-3, Olympus) is used as an excitation source and is passed through the PSG unit for generating the input polarization states. The PSG unit consists of a horizontally oriented fixed linear polarizer P1 and a rotatable achromatic quarter waveplate (QWP1, AQWP05M-600, Thorlabs, USA) mounted on a computer controlled rotational mount (PRM1/M-27E, Thorlabs, USA). The PSG-emerging light is then focused to an annular shape at the sample site using a dark-field condenser (Olympus U-DCD, NA = 0.92). The sample-scattered light is collected by the microscope objective (MPlanFL N, NA = 0.8), passed through the PSA unit (for the analysis of the polarization state of the scattered light) and is then relayed for spectrally resolved signal detection. The dark-field arrangement facilitates detection of exclusively the sample-scattered light (scattering spectra). The PSA unit essentially comprises of the same polarization components with a fixed linear polarizer (P2, oriented at vertical position) and a computer controlled rotating achromatic quarter waveplate (QWP2), but positioned in a reverse order. Spectrally resolved signal detection is performed by a spectrometer (HR 2000, Ocean optics, USA). This enables recording of scattering spectra (λ = 400–700 nm) with a resolution ~1.5 nm. The spectra were recorded with an acquisition time of ~0.05s and for each polarization resolved measurements averaging were done over 100 spectra.

The salient advantages of the experimental system are worth a brief mention. First of all, the issues pertaining to polarization measurements over broad spectral range and the polarization transformation due to the high NA imaging geometry are tackled by determining the actual experimental *W*(λ) and *A*(λ) matrices (over λ = 400–700 nm). Secondly, this measurement scheme is independent of the polarization response of the spectrometer (detector) and the source, since it uses fixed linear polarizers at both the excitation (P1-horizontal) and the detection (P2-vertical) end. Finally, the entire experimental system is automated using Labview for easier and faster data acquisition. The system is capable of recording the spectroscopic Mueller matrices by using the spectrometer.

### Plasmonic sample preparation

Gold (Au) nanorods were synthesized as described in the literature by Babak *et al*.^37^. All the chemicals like tetrachloroauric acid (HAuCl_4_.3H_2_O), L-ascorbic acid, silver nitrate (AgNO_3_) sodium borohydride (NaBH_4_) and CTAB (cetyltrimethylammonium bromide) were procured from Sigma-Aldrich. The entire reaction was carried out in two parts at room temperature. In the first part, we synthesized the seed solution by mixing both 5 mL of 0.2 MolCTAB and 5 ml of 0.5 mMol HAuCl_4_ solutions. Then 0.6 ml of ice-cold 0.01 Mol NaBH_4_ was added to the above mixture under continuous stirring for 5 min which produces seed solution with nanoparticles of size less than 10 nm. The second part is about the preparation of growth solution where we would like to grow Au nanorods from the existing nanoparticles in seed solution. Growth solution is prepared by mixing 5 ml of 0.2 Mol CTAB, 5 ml of 1 mMol HAuCl_4_, 150 μl of 0.0064 Mol AgNO_3_, and 85 μl of 0.0788 Mol L-ascorbic acid. To obtain the required size of Au nanorods, 36 μl of seed solution was mixed with the growth solution under continuous stirring for 1 hour. Au nanorod images were recorded by a Zeiss SUPRA 55VP-Field Emission Scanning Electron Microscope (FE-SEM) by spin coating the Au nanorod solution on a clean silicon (100) substrate. For the Mueller matrix spectroscopic and imaging studies, the samples were diluted as required and then fixed on a glass cover slip by spin coating (Apex Instrumentation, spinNXG - P1), which was then kept at the sample stage of the dark-field microscopic arrangement.

## Additional Information

**How to cite this article**: Chandel, S. *et al*. Complete polarization characterization of single plasmonic nanoparticle enabled by a novel Dark-field Mueller matrix spectroscopy system. *Sci. Rep*. **6**, 26466; doi: 10.1038/srep26466 (2016).

## Supplementary Material

Supplementary Information
