# Supplementary material for: Complete polarization characterization of single plasmonic nanoparticle enabled by a novel Dark-field Mueller matrix spectroscopy system
Source: Sci Rep. 2016 May 23;6:26466. doi: 10.1038/srep26466 (PMC4876463; doi:10.1038/srep26466)
Supplement: Supplementary Information [file srep26466-s1.pdf]

Supporting Information

Complete polarization characterization of single plasmonic nanoparticle enabled by a novel Dark-field Mueller matrix spectroscopy system

Shubham Chandel, Jalpa Soni, Subir K. Ray, Anwesh Das, Anirudha Ghosh, Satyabrata Raj and Nirmalya Ghosh**

The steps of the eigenvalue calibration method which is used for the calibration of the developed Dark-field Mueller matrix spectroscopic microscopy system are outlined in this supporting information file.

Eigenvalue Calibration method

The actual experimental polarization state generator $W(\lambda)$ and analyzer $A(\lambda)$ matrices are determined using measurements on a set of ideal calibrating samples (pure diattenuators (polarizers) and retarders (waveplates)), as follows. Sixteen (4×4) set of spectral measurements are performed separately with the calibrating sample (s) in place (B) and without any sample (blank) (B_0). These are related as

$$B = AMW; \quad B_0 = AW \tag{1}$$

Here, $M$is the unknown Mueller matrix of the calibrating sample. Two set of matrices C and $C^/$ are then constructed such that the former is independent of A and the latter is independent of W

$$C = B_0^{-1}B = W^{-1}MW; \; C^/ = BB_0^{-1} = AMA^{-1} \tag{2}$$

Using equation (2), eigenvalues of the Mueller matrix M of the calibrating sample can be determined from the eigenvalues of either of the experimental matrices C or $C^/$ ($M, C, C^/$have same eigenvalues). The experimental Mueller matrix M is constructed from the determined eigenvalues as follows. The general Mueller matrix of a diattenuating retarder (can be either a pure diattenuator or a retarder also) calibrating sample has the following form

$$M = \begin{bmatrix} 1 & -\cos 2\psi & 0 & 0 \\ -\cos 2\psi & 1 & 0 & 0 \\ 0 & 0 & \sin 2\psi \cos\Delta & \sin 2\psi \sin\Delta \\ 0 & 0 & \sin 2\psi \sin\Delta & \sin 2\psi \cos\Delta \end{bmatrix} \quad (3)$$

Where Δ and ψ are the conventional ellipsometric parameters for the diattenuating retarder with τ being its transmittance. This Mueller matrix M has two real and two complex eigenvalues

$$\lambda_{R1} = 2\tau\cos^2\psi \qquad \lambda_{R2} = 2\tau\sin^2\psi \qquad \lambda_{C1} = \tau\sin 2\psi e^{-i\Delta} \qquad \lambda_{C2} = \tau\sin 2\psi e^{i\Delta} \quad (4)$$

The transmittance τ and the ellipsometric parameters Δ, ψ can thus be obtained from the eigenvalues of M (as determined from the experimental C or $C^{/}$ matrices, Eq. 2) as

$$\tau = \frac{(\lambda_{R1} + \lambda_{R2})}{2}, \qquad \psi = \tan^{-1}\sqrt{\frac{\lambda_{R1}}{\lambda_{R2}}} \qquad \text{and} \qquad \Delta = \log\sqrt{\frac{\lambda_{C2}}{\lambda_{C1}}} \quad (5)$$

The Mueller matrix M of the reference sample can then be constructed using Eqs. 3 and 5. [1,3] Once the Mueller matrix M is determined, the generator W and the analyzer A matrices can be determined using Eq. 2. The W matrix is determined by solving the following equation.

$$MW - WC = 0 \quad (6)$$

In order to solve the above equation, a linear operator K is formed such a way that W is the only eigenvector associated with the null eigenvalue (satisfying K $W_{16\times 1} = 0$). [1,3]

Note that K has all different eigenvalues from zero except λ_1, which is supposed to be null and practically as close as zero ($0=\lambda_1 << \lambda_2 < \lambda_{3\ldots}\ \lambda_{16}$). The smallest eigenvalue of the matrix (corresponding to the obtained eigenvector ($W_{16\times 1}$)) K is reshaped in 4 × 4 matrix to obtain the generator matrix W. With the W matrix in hand, the analyzer matrix A can be determined as

$$A = B_0 W^{-1} \quad (7)$$

The exact nature of the system $W(\lambda)$ and analyzer $A(\lambda)$ matrices and their wavelength dependence was determined by performing measurements on two different types of calibrating reference samples. We used linear polarizer (pure diattenuator) and broadband quarter waveplate (pure retarder over λ = 400 – 700 nm) as reference samples. Once, the experimental $W(\lambda)$ and $A(\lambda)$ matrices are determined, they can be used to determine Mueller matrices $M(\lambda)$ of any unknown sample using Equation. 2 of the manuscript.

Scattering angle dependence of diattenuation and retardance

In the Fig. S1 below, the dependence of the Mueller matrix-derived (using Eq. 3 and 4 of the manuscript) diattenuation d and retardance δ parameters on the polar scattering angle θ is shown. The Mueller matrices were computed for a preferentially oriented nano-rod of the size (40×14 nm) as a function of the forward scattering angles. For the angular variation of the d-parameter, the wavelength is chosen to be λ = 650 nm, corresponding to the peak of the longitudinal dipolar plasmon resonance. For the angular variation of the δ –parameter, on the other hand, λ is chosen to be 550 nm, corresponding to the spectral overlap region of the two orthogonal dipolar plasmon resonance (where the magnitude of δ attains its maximum value).

As expected from the predictions of Eq. 5, in the dipolar scattering approximation, the δ parameter is relatively insensitive to the scattering angle. In contrast, the d-parameter varies with θ, due to the presence of the he $cos^2\theta$ factor associated with dipolar scattering.

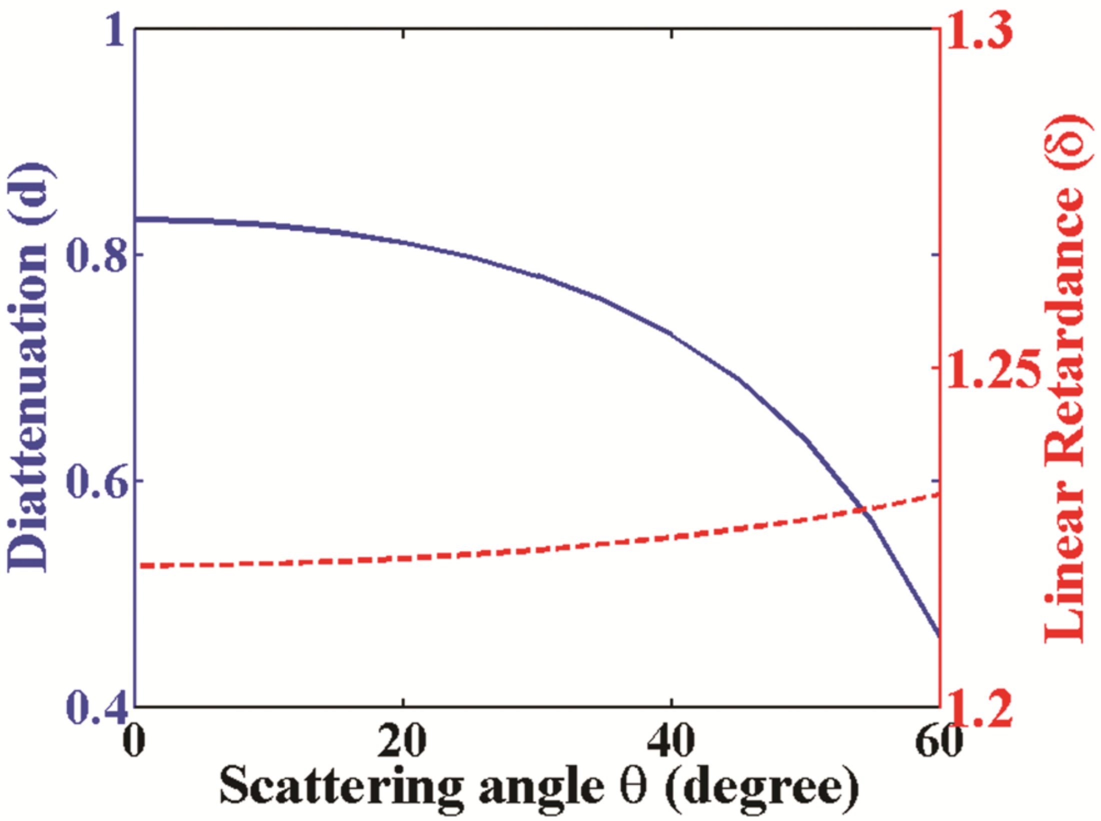

Figure S1: The (theoretically computed) Mueller matrix-derived scattering angle (θ) variation of the linear retardance δ (right axis, red dotted line) and linear diattenuation d (left axis, blue solid line) parameters for the preferentially oriented Au-nanorods (40×14 nm). While for the d parameter, the wavelength is chosen to be λ = 650 nm (corresponding to the peak of the longitudinal dipolar plasmon resonance plasmon), for the δ parameter, λ is chosen to be 550 nm (corresponding to the spectral overlap region of the two orthogonal dipolar plasmon resonance).

References:

1. B. Laude-Boulesteix, A. De Martino, B. Drévillon, L. Schwartz, *Appl. Opt.* **2004,**43(14), 2824–2832.
2. A. De Martino, E. Garcia-Caurel, B. Laude, and B. Drévillon, "General methods for optimized design and calibration of Mueller polarimeters," *Thin Solid Films*, **2004,** 455−456, 112-119.
3. F. Stabo-Eeg, PhD Thesis, Norwegian University of Science and Technology, **2009**.
